# Climate Change–Induced Stress Reduce Quantity and Alter Composition of Nectar and Pollen From a Bee-Pollinated Species (*Borago officinalis*, Boraginaceae)

**DOI:** 10.3389/fpls.2021.755843

**Published:** 2021-10-11

**Authors:** Charlotte Descamps, Muriel Quinet, Anne-Laure Jacquemart

**Affiliations:** Earth and Life Institute – Agronomy, UCLouvain, Louvain-la-Neuve, Belgium

**Keywords:** water stress, temperature rise, nectar, pollen, polypeptides, amino acids, sugars, floral resource

## Abstract

In temperate ecosystems, elevated temperatures, and drought occur especially during spring and summer, which are crucial periods for flowering, pollination, and reproduction of a majority of temperate plants. While many mechanisms may underlie pollinator decline in the wake of climate change, the interactive effects of temperature and water stress on the quantity and quality of floral nectar and pollen resources remain poorly studied. We investigated the impact of temperature rise (+3 and +6°C) and water stress (soil humidity lower than 15%) on the floral resources produced by the bee-pollinated species *Borago officinalis*. Nectar volume decreased with both temperature rise and water stress (6.1 ± 0.5 μl per flower under control conditions, 0.8 ± 0.1 μl per flower under high temperature and water stress conditions), resulting in a 60% decrease in the total quantity of nectar sugars (mg) produced per flower. Temperature rise but not water stress also induced a 50% decrease in pollen weight per flower but a 65% increase in pollen polypeptide concentration. Both temperature rise and water stress increased the total amino acid concentration and the essential amino acid percentage in nectar but not in pollen. In both pollen and nectar, the relative percentage of the different amino acids were modified under stresses. We discuss these modifications in floral resources in regards to plant–pollinator interactions and consequences on plant pollination success and on insect nutritional needs.

## Introduction

Drought combined with heatwaves are expected to increase in both frequency and intensity under climate change (IPCC, 2018; Spinoni et al., 2018). Climate-induced abiotic stresses can trigger physiological and morphological changes in flowering plants (Scaven and Rafferty, 2013; Pandey et al., 2015; Lamaoui et al., 2018), especially during plant reproductive phases (Barnabás et al., 2008; Scheepens et al., 2018). Plant–pollinator interactions rely on floral traits and resources (Michener, 2007), which may be disrupted by plant stress responses. Given that most angiosperms depend on insects for reproduction (Ollerton et al., 2011; IPBES, 2016), climate-driven alterations in plant–pollinator interactions could have severe ecological and economic consequences globally (Potts et al., 2010; Settele et al., 2016). The major pollinators in temperate regions are bees (Hymenoptera, *Anthophila*), which exclusively depend on pollen and nectar resources for their survival (Michener, 2007; Brodschneider and Crailsheim, 2010; Nicolson, 2011; Vaudo et al., 2015; IPBES, 2016).

Floral resources for pollinators include nectar and pollen. Nectar consists in a sweet aqueous solution and represents the major energy source for pollinating insects. In addition to sugars (mainly glucose, fructose, and sucrose), nectar also contains amino acids, minerals, and secondary metabolites in lower quantities (Nicolson and Thornburg, 2007; Parachnowitsch et al., 2019). After sugars, amino acids are the most abundant nutrients in nectar (Heil, 2011). Amino acid composition and concentration differ depending on plant species and environmental conditions (Baker, 1977; Gardener and Gillman, 2002; Suni et al., 2020; Venjakob et al., 2020).

Pollen consists mostly of proteins and lipids and is a major nutrient source for larval growth and development and for adult immunocompetence, reproduction, and longevity (Roulston and Cane, 2000; Pasquale et al., 2016; Moerman et al., 2017; Moquet et al., 2017). As bees cannot synthesize essential amino acids *de novo*, they rely exclusively on pollen for these essential compounds (de Groot, 1952; Nicolson, 2011). Protein content is highly variable among plant species, ranging from 2.5 to 60% of dry mass (Roulston and Cane, 2000; Vanderplanck et al., 2014b). Protein content alone does not define the nutritional value of pollen for bees, as it also depends on the amino acid composition.

The combined effects of water stress and temperature rise on pollen and nectar resources remain poorly studied (Scaven and Rafferty, 2013; Forrest, 2016; Borghi et al., 2019). These abiotic constraints can individually alter both the composition and quantity of floral resources, but responses are often species- and context-dependent (Scaven and Rafferty, 2013; Descamps et al., 2021b). Nectar is a floral resource with a dynamic secretion, varying in volume and composition with environmental conditions, flower age, and position (Corbet, 2003; Nicolson and Thornburg, 2007; Parachnowitsch et al., 2019). Nectar volume decreases with water stress and supra-optimal temperatures in many species (Scaven and Rafferty, 2013; Descamps et al., 2021b). Supra-optimal temperatures are temperature above the temperature optimum for plant growth and reproduction, which is species-specific (Korres et al., 2016). The effects of abiotic stresses on the sugar concentrations in nectar are less clear: it increases or remains stable depending on the species and abiotic conditions (Phillips et al., 2018; Takkis et al., 2018; Brunet and Van Etten, 2019; Descamps et al., 2020).

Pollen is particularly sensitive to abiotic stresses and all stages of pollen development are affected, leading to a reduced production of pollen grains and a lower pollen viability under stressful conditions (Koti et al., 2005; Mesihovic et al., 2016; Pacini and Dolferus, 2019). Temperature rise and water stress which can impair photosynthesis process, may negatively affect sucrose metabolism and transport (Prasad et al., 2008; Sehgal et al., 2019). The photosynthesis changes reduce starch and lipid accumulation in pollen grains, leading to a decrease of pollen viability (Pressman et al., 2002; Borghi et al., 2019). Modifications of pollen composition can negatively impact the nutritive value for pollinators, potentially reducing their foraging performance, health and survival (Vanderplanck et al., 2014b; Roger et al., 2017). Anther dehiscence at anthesis can also be impaired by stresses, limiting pollen dispersal and transfer (Smith and Zhao, 2016). All of these changes can decrease plant pollination and consequently, the plant reproductive success through fruit and seed sets (Snider and Oosterhuis, 2011; Su et al., 2013; Sehgal et al., 2019).

In this study, we quantified floral nectar and pollen modifications due to temperature rise (+3 and +6°C) and water stress in a highly attractive bee-pollinated species, *Borago officinalis* (Boraginaceae). The reproductive development of *B*. *officinalis* is impaired by temperature rise and water stress (Descamps et al., 2018), and here we study potential impacts of these stresses on traits linked to pollinator attractiveness and nutritional requirements. To our knowledge, this is the first study of its kind that examines the interaction of temperature and water stress on both the quantity and composition (polypeptide, amino acid, and sugar concentrations) of floral resources.

## Materials and Methods

### Plant Material and Growth Conditions

*Borago officinalis* (Boraginaceae) is an annual, entomophilous bee-pollinated plant species. The flowering period extends from June to September in temperate areas, and about 100 flowers are produced per plant. This species produces abundant floral resources (about 5 μl nectar and 12,700 pollen grains), which makes it highly attractive to pollinators (Thom et al., 2016; Descamps et al., 2018). The anthers and nectaries are easily accessible on the surface of the corolla.

*Borago officinalis* seeds were provided by Semailles nursery (Faulx-les-Tombes, Belgium). Seedlings at the three-leaf stage were transplanted into 2-L pots filled with a 1:1 (v/v) mix of sand (size 0/5, M PRO, Netherlands) and universal peat compost (DCM, Amsterdam, Netherlands) and grown in the glasshouses at the University campus (SEFY platform, Louvain-la-Neuve, Belgium). Plants were watered daily with rainwater. Treatments were applied at floral transition, 4 weeks after sowing. At this stage, bolting occurred, flowering stems developed, and the first floral buds were visible. Plants were subjected to three temperature regimes (21, 24, and 27°C) and two watering regimes (well-watered vs. water-stressed). The well-watered plants received daily watering (soil humidity about 30%), whereas the water-stressed plants were watered twice a week (soil humidity lower than 15%, Descamps et al., 2018). Soil water content was quantified with a ProCheck sensor handheld reader (Decagon Devices, Pullman, United States). In total, six treatments were applied to 12 plants per treatment: 21°C well-watered (21WW), 21°C water- stressed (21WS), 24°C well-watered (24WW), 24°C water-stressed (24WS), 27°C well-watered (27WW), and 27°C water-stressed (27WS). Seventy-two plants were monitored in three growth chambers under three temperature regimes (day/night): 21°C/19°C, 24°C/22°C, and 27°C/25°C. The photoperiod was 16 h light:8 h dark, and relative humidity was maintained at 80 ± 10%. Each growth chamber was divided into two parts to accommodate two watering regimes. 21WW was considered the “control” treatment with optimal temperature growth and watering regime, while 27WS was considered the most stressful treatment, with combined high temperature and water stress conditions (Descamps et al., 2018). Growth chamber experiments lasted 5 weeks. Water stress was applied after 1 week of acclimation to the growth chambers (this week was considered week 0). Experiments were performed in June–July 2020.

### Nectar Sampling and Chemical Analyses

During weeks 2 and 3, nectar was collected with 10-μl glass capillary tubes (Hirschmann Laborgeräte, Eberstadt, Germany). The nectar volume per flower was estimated for seven flowers per treatment by measuring the length of the nectar column in the capillary tube. The total sugar concentration (C,°Brix, g sucrose/100 g solution) was measured on the same flowers with a low-volume hand refractometer (Eclipse handheld refractometer; Bellingham and Stanley, Tunbridge Wells, United Kingdom). Nectar sugar content per flower (*s*, mg) was calculated as *s* = 10 × *d* × *v* × *C*, where d is the density of a sucrose solution at concentration *C* (*d* = 0.0037921 × *C* + 0.0000178 × *C*^2^ + 0.9988603) and *v* is nectar volume (ml) (Prys-Jones and Corbet, 1991).

Ten samples of 10 μl of nectar were taken per treatment (from a minimum of two flowers from two different plants) and analyzed for sugar composition and amino acid content. The sugar composition of the nectar was determined using gas chromatography–flame ionization detection (GC-FID, Thermo Scientific, Germany) using a Restek RTX5-MS column (30 × 0.25 mm i.d. 0.25 μm) as described by Somme et al. (2016). The nectar extracted from the capillaries was weighed (Sartorius-Basic scale 0.1 mg, Germany) and 1 ml water was added. Internal standard (50 μl of a 1,000 ppm aqueous mannitol solution) was added to 50 μl diluted sample. This mixture was then dried under N_2_ before forming trimethylsilyl (TMS) derivatives [using hydroxylamine hydrochloride and hexamethyldisilazide (HMDS) in pyridine]. A 1-μl volume of TMS derivatives was injected into GC-FID and separated via a Restek RTX5-MS column (30 × 0.25 mm i.d. 0.25 μm) in split mode (1/10). Nitrogen at 2 ml/min was used as carrier gas. The injector and detector were set to 270 and 300°C, respectively. The temperature program started at 105°C for 4 min, before increasing at a rate of 15°C/min to 280°C. This temperature was maintained for 20 min. All analytical measures were performed in duplicate.

Amino acids were quantified using high performance liquid chromatography (HPLC), in duplicate after derivatization using phthaldialdehyde (OPA) reagent in combination with 9-fluorenylmethyl chloroformate (FMOC, Meussen et al., 2014; Aubert et al., 2021). The nectar extracted from the capillaries was weighed (Sartorius-Basic scale, Germany) and 100 μl of an aqueous solution of 25 μM norvaline (used as an internal standard) was added. As no acid hydrolysis took place, we could not measure the tryptophan content. The determination of cysteine and methionine required a prior oxidation step (H_2_O_2_/formic acid, 110°C for 18 h). A double derivatization process was performed in pre-columns using (i) 2-mercaptoethanol 4% + 25 mg OPA dissolved in 0.5 ml methanol in a total volume of 5 ml borate buffer pH 10.4 [for detection of all amino acids except proline (PRO) and hydroxy-proline (OH-PRO)] and (ii) FMOC 0.25% in acetonitrile (for detection of cyclic amino acids: PRO and OH-PRO). Hydroxy-proline is an amino acid derived from proline by hydroxylation. Samples were injected on a Zorbax Eclipse Plus column (Agilent; 3.5 μm particle size; 150 × 21 mm) maintained at 40°C. The mobile phase was composed of (A) phosphate buffer 40 mM pH 8.4 and (B) acetonitrile/methanol/water (45:45:10 *v/v/v*) at a flow rate of 0.42 ml/min (100% A–0% B 0.5 min; progressive increase from 0 to 57% B 0.5–25 min). OPA-derivatized and FMOC-derivatized amino acids were, respectively, detected at 340 and 266 nm excitation and 450 and 338 nm emission wavelengths.

### Pollen Sampling and Chemical Analyses

During weeks 2 and 3, pollen was collected from at least 10 flowers per treatment (minimum of two different plants), by squeezing and opening anthers with pliers over a microfuge tube. We estimated pollen production (mg) per flower by dividing the weight of each sample by the number of flowers from which pollen was collected.

Polypeptide content (molecular weight > 10 kDa) of pollen was determined from 5 mg dry pollen in triplicate for each treatment following the method described in Vanderplanck et al. (2014a). Total polypeptides were quantified using the bicinchoninic acid (BCA) Protein Assay Kit (Pierce, Thermo Scientific), with BSA as standard.

Amino acid profile and concentrations were determined from 2 mg pollen in triplicate after acid hydrolysis (with or without prior oxidation) and derivatization using OPA reagent in combination with FMOC (Meussen et al., 2014; Aubert et al., 2021). For acid hydrolysis, samples were exposed to 150 μl 6 M hydrochloric acid containing 1% (w/v) of phenol and incubated for 18 h at 110°C. For acid hydrolysis with prior oxidation, 50 μl performic acid reagent was added to the samples. The reagent consisted of 10 mg phenol crystal in 10 ml water with 1 ml 33% H_2_O_2_ and 9 ml 88% formic acid. After addition of the reagent, tubes were incubated for 3 h at 4°C, and 8 mg metabisulfite was then added to decompose the performic acid. Tubes were then stirred for 1.5 s to liberate any SO_2_ produced. Derivatization and quantification were as described for the nectar samples. The essential amino acids were determined by de Groot (1952) for *Apis mellifera* and include*:* arginine, histidine, isoleucine, leucine, lysine, methionine, phenylalanine, threonine, tryptophan, and valine. This information is not known for wild bee species (Kriesell et al., 2017; Woodard and Jha, 2017; Nikkeshi et al., 2021).

All chemical analyses were performed in the MOCA platform and PEPA lab (UCLouvain, Louvain-la-Neuve, Belgium).

### Statistical Analyses

Normality of the data was visually assessed using quantile–quantile plots. Linear mixed models and analysis of variance (type II) were performed using a significance level of *p* < 0.05 to evaluate the effects of temperature, water stress, and their interaction. For nectar volume, sugar concentration, and pollen dry weight, analyses of variance (type II) were performed. Linear mixed models were fit including the two fixed factors, their interaction (temperature × water) and a random effect for sampling number due to repeated measurements on the same sample (sugar proportion, polypeptide concentration, and amino acid concentration). Results for all amino acids were presented as relative differences compared with the control treatment (21WW) for each amino acid. The relative difference was obtained by subtracting the value of the 21WW treatment concentration from the value of each treatment concentration, divided by the value of the 21WW treatment concentration. Amino acids are only shown when their concentrations significantly varied with temperature and/or water stress. Concentrations for each amino acid for each treatment with statistical results are available in Supplementary Material. To obtain a global view of the influence of temperature rise and water stress on all measures, a principal component analysis (PCA) was performed. All analyses were performed with R 3.6.1 (R Core Team, 2020), using the package *car* for *F* tests, the package *lme4* for linear mixed models, the package *FactomineR* for PCA and packages *ggplot2* and *yarr* for plots. Data are presented as mean ± standard error (SE).

## Results

### Floral Resources Were Affected by Temperature Rise and Water Stress

**FIGURE 1:**
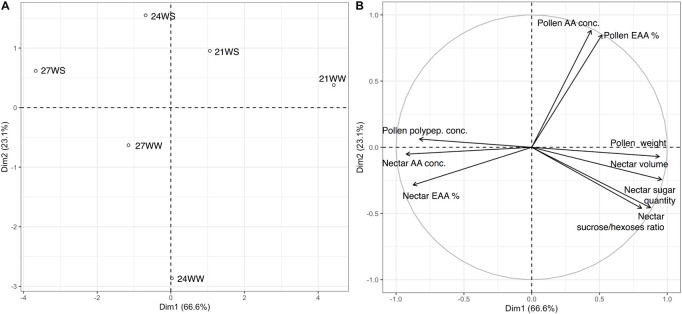
Principal component analysis (PCA) of nectar and pollen resource measurements from *Borago officinalis* plants grown under different temperature regimes (21, 24, and 27°C) and watering regimes (WS, water-stressed; WW, well-watered): **(A)** treatment centroids; **(B)** variable loadings of PCA 3 weeks after stress induction. Polypep., polypeptides; Conc., concentration; %, percentage; EAA, essential amino acids.

We performed a principal component analysis of all nectar and pollen measurements according to the different treatments. The first two principal components explained 89.6% of the variance, with axis 1 accounting for 74.8% of the variance (Figure 1). The difference between the control treatment (21WW) and the most stressful treatment (27WS) was evident along axis 1. Floral resource quantities (nectar volume, and pollen dry weight) were higher for plants grown at 21WW than for plants grown at 27WS, while pollen polypeptide concentration and nectar amino acid concentration were higher for plants grown at 27WS than for plants grown at 21WW. Axis 2 discriminated plants grown at 21WS and 24WS from plants grown at 24WW according to the amino acid concentration of pollen.

### The Influence of Temperature Rise and Water Stress on Nectar

#### Nectar Volume and Sugar Concentration

**FIGURE 2:**
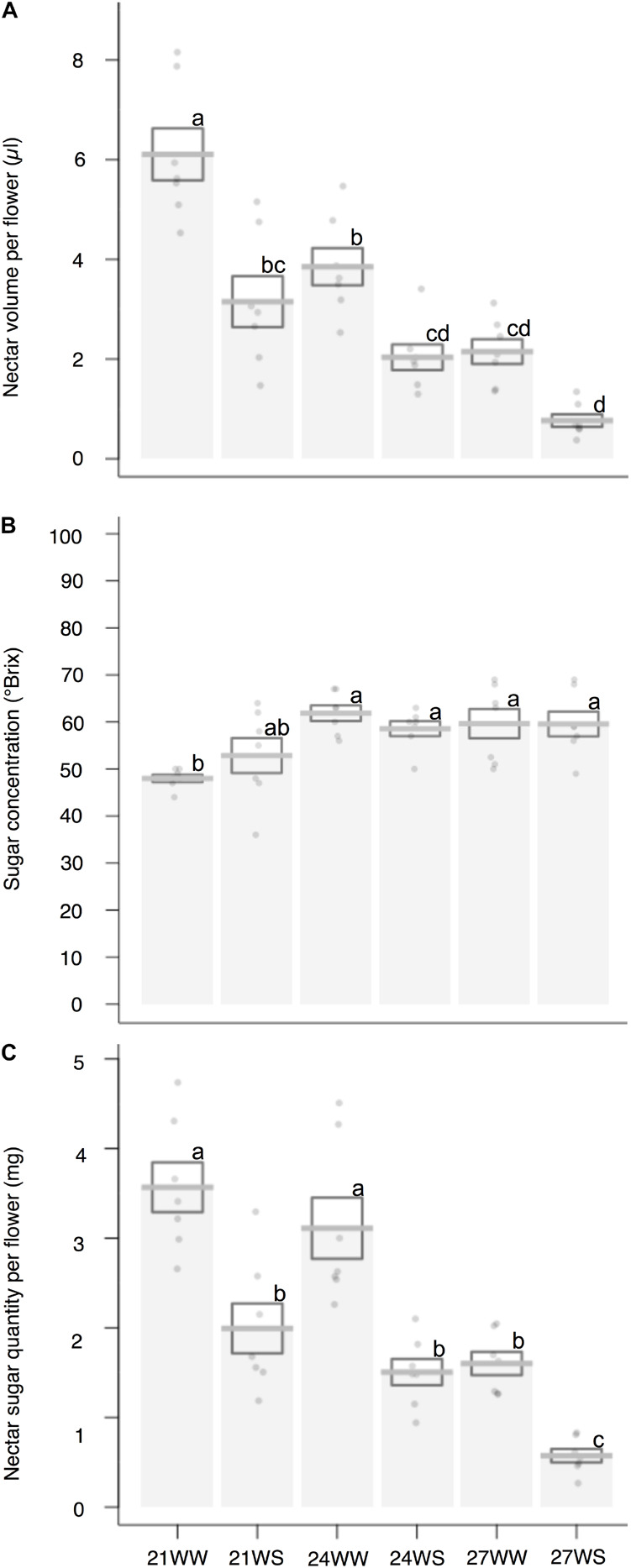
Effects of temperature and water stress on nectar of *B*. *officinalis*, 3 weeks after stress induction: **(A)** nectar volume per flower (μl); **(B)** nectar sugar concentration (°Brix); **(C)** nectar sugar quantity per flower (mg). *N* = 7 plants. Data are means ± SE. Data points followed by different letters for the same parameter are significantly different at *P* < 0.05 among treatments. 21, 21°C; 24, 24°C; and 27, 27°C; WW, well-watered; WS, water-stressed.

Nectar volume decreased (6.1 ± 0.5 μl per flower under 21WW, 0.8 ± 0.1 μl per flower under 27WS) with temperature rise (*F*_2_,_36_ = 37.05, *P* < 0.001) and water stress (*F*_1_,_36_ = 46.46, *P* < 0.001; Figure 2A). Nectar sugar concentration increased (50 ± 3°Brix under 21°C, 60 ± 3°Brix under 27°C) with temperature rise (*F*_2_,_36_ = 9.92, *P* < 0.001) but was not affected by water stress (*F*_1_,_36_ = 0.06; *P* = 0.80; Figure 2B). In consequence, nectar sugar quantity per flower was reduced by temperature rise (*F*_2_,_36_ = 29.14, *P* < 0.001) and water stress (*F*_1_,_36_ = 56.36, *P* < 0.001; Figure 2C), resulting in a six-fold reduction in nectar sugar quantity for plants grown at 27WS (0.57 ± 0.08 mg) compared to plants grown at 21WW (3.57 ± 0.28 mg).

#### Nectar Sugar Composition

Nectar composition was dominated by sucrose regardless of the treatment applied, with sucrose/hexoses ratio higher than 1 (Table 1). The proportion of fructose was higher compared to the proportion of glucose. We then determined the effects of both stresses on nectar sugar composition: we observed that water stress decreased the sucrose content by 4–5% and increased the glucose and fructose content by 2–3% (Table 1), resulting in a decrease of the sucrose/hexoses ratio. Temperature rise did not significantly modify any of the sugar proportions.

**TABLE 1:** Effects of temperature and water stress on nectar sugar composition of *Borago officinalis* 3 weeks after stress induction.

Treatment^1^	21WW	21WS	24WW	24WS	27WW	27WS	Temp^2^	Water	Temp:Water
Fructose (%)	25.4 ± 0.6	27.6 ± 0.6	26.7 ± 0.9	28.4 ± 0.8	26.5 ± 0.9	29.8 ± 0.9	*F*_2,18_ = 2.05; *P* = 0.16	*F*_1,18_ = 12.03; *P* = 0.003	*F*_2,18_ = 0.52; *P* = 0.6
Glucose (%)	17.2 ± 0.3	19.3 ± 0.5	17.9 ± 0.6	19.8 ± 0.9	18.3 ± 0.7	20.7 ± 0.5	*F*_2,18_ = 1.96; *P* = 0.17	*F*_1,18_ = 15.94; *P* < 0.001	*F*_2,18_ = 0.08; *P* = 0.92
Sucrose (%)	57.3 ± 0.8	53.2 ± 1.1	55.3 ± 1.5	51.8 ± 1.7	55.2 ± 1.6	49.5 ± 1.4	*F*_2,18_ = 2.09; *P* = 0.15	*F*_1,18_ = 14.28; *P* = 0.001	*F*_2,18_ = 0.3 *P* = 0.75
Sucrose/hexoses ratio	1.35 ± 0.05	1.14 ± 0.05	1.25 ± 0.07	1.09 ± 0.07	1.24 ± 0.08	0.99 ± 0.05	*F*_2,18_ = 1.84; *P* = 0.19	*F*_1,18_ = 13.89; *P* = 0.002	*F*_2,18_ = 0.23; *P* = 0.79

*^1^N = 3 (replicates). Data are mean ± SE. 21, 21°C; 24, 24°C; 27, 27°C; WW, well-watered; WS, water-stressed.*

*^2^Two-way ANOVA results, testing for the main and the interactive effect of temperature (Temp.) and water treatments.*

#### Nectar Amino Acid Content

**FIGURE 3:**
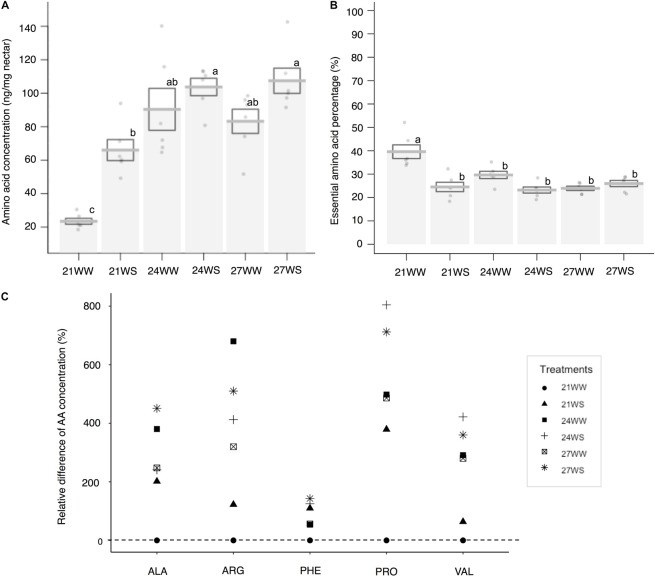
Effects of temperature rise and water stress on nectar amino acid content of *B*. *officinalis*, 3 weeks after stress induction: **(A)** total nectar amino acid concentration (ng/mg nectar); **(B)** essential amino acid percentage compared to total concentration (%); **(C)** relative difference in amino acid concentration (%). *N* = 3 plants (replicates). **(A,B)** Data are mean ± SE; data points followed by different letters for the same parameter are significantly different at *P* < 0.05 among treatments; **(C)** data are mean and relative values compared with 21WW. 21, 21°C; 24, 24°C; and 27, 27°C; WW, well-watered; WS, water-stressed. Only significant changes in amino acid concentrations are shown: ALA, alanine; ARG, arginine; PHE, phenylalanine; VAL, valine; PRO, proline.

The total nectar amino acid content was on average 0.19 μg per flower at 21WW (control). The three most abundant amino acids in percentage in nectar of plants grown at 21WW were proline (22.7%), histidine (10.9%), and glutamine (8.8%). The total amino acid concentration increased with temperature rise (*F*_2_,_12_ = 17.1, *P* < 0.001) and water stress (*F*_1_,_12_ = 10.4, *P* = 0.007; Figure 3A). Increase of total amino acid concentration was explained by the increase of five amino acid concentrations: alanine, arginine, phenylalanine, proline, and valine (Figure 3C and Supplementary Table 1). Temperature induced increases in the concentrations of arginine, alanine and valine. Water stress induced increases in the concentration of phenylalanine. Both types of stress increased the concentration of proline. The three most abundant amino acids in nectar of plants grown at 27WS were proline (40.1%), serine (10.5%), and glutamine (8.6%). The concentrations of the other amino acids were not affected by the applied abiotic stresses (Supplementary Table 1). The proportion of essential amino acids was higher in nectar from plants grown at 21WW compared to the other treatments (Figure 3B and Supplementary Table 1).

### The Influence of Temperature Rise and Water Stress on Pollen

We quantified the modification of pollen quantity and composition (polypeptides and amino acid profil) induced by temperature rise and water stress.

#### Pollen Quantity

**FIGURE 4:**
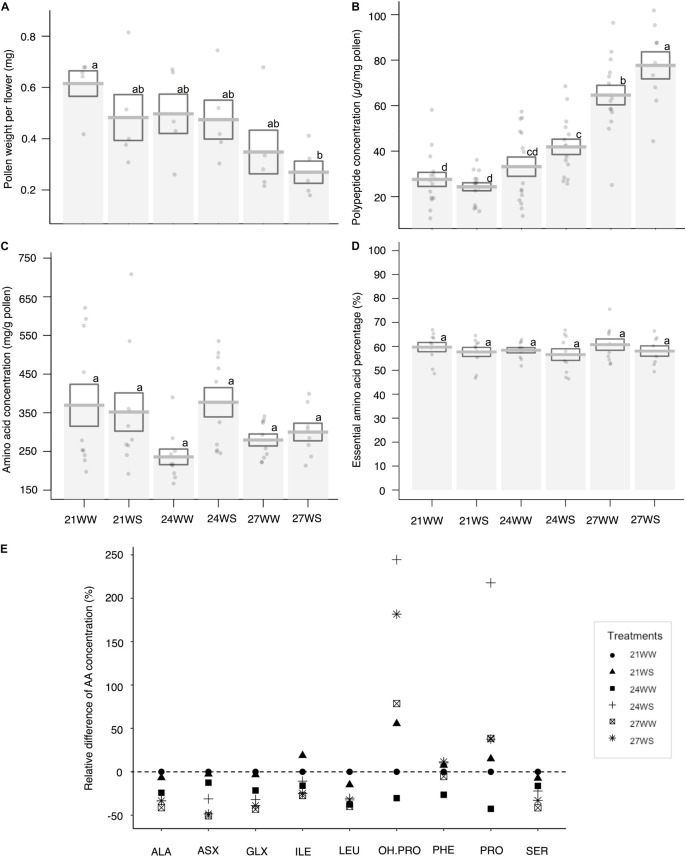
Effects of temperature rise and water stress on pollen quantity and composition of *B*. *officinalis*, 3 weeks after stress induction: **(A)** pollen weight per flower (mg); **(B)** total polypeptide concentration (μg/mg pollen); **(C)** total nectar amino acid concentration (ng/mg nectar); **(D)** essential amino acid percentage compared to total concentrations (%); **(E)** relative difference of amino acid concentrations (%). **(A,B)** *N* = 5 plants, **(C–E)** *N* = 3 plants. **(A–D)** Data are mean ± SE; data points followed by different letters for the same parameter are significantly different at *P* < 0.05 among treatments; **(E)** data are mean and relative values compared with 21WW. 21, 21°C; 24, 24°C; and 27, 27°C; WW, well-watered; WS, water-stressed. Only significant changes in amino acid concentrations are shown: ALA, alanine; ASX, asparagine + aspartic acid; GLX, glutamine + glutamic acid; ILE, isoleucine; LEU, leucine; OH.PRO, hydroxyproline; PHE, phenylalanine; PRO, proline; SER, serine.

Pollen weight per flower decreased with temperature stress (*F*_2_,_24_ = 5.95, *P* = 0.007) but was not affected by water stress (*F*_1_,_24_ = 1.75, *P* = 0.2; Figure 4A). Pollen weight was halved between plants grown at 21°C (0.61 ± 0.05 mg) and 27°C (0.35 ± 0.09 mg).

#### Pollen Polypeptides

The polypeptide concentration increased by almost 200% between control conditions and the most stressful conditions (27.6 ± 4.1 μg/mg pollen at 21WW vs. 77.7 ± 6.6 μg/mg pollen at 27WS). Pollen polypeptide concentration increased with temperature (*F*_2_,_78_ = 69.29, *P* < 0.001; Figure 4B) but was not affected by water stress (*F*_1_,_78_ = 3.25, *P* = 0.08). The average quantity of polypeptides in pollen per flower remained constant among treatments (18.6 μg/flower at 21WW compared to 18.8 μg/flower at 27WS).

#### Pollen Amino Acid Content

Under control conditions (21WW), flowers produced on average 0.23 mg of total pollen amino acids. For plants grown at 21WW, the three most abundant amino acids in pollen were lysine (16.2%), arginine (11.2%), and glycine (10.7%).

Total amino acid concentrations did not significantly differ with temperature rise (*F*_2_,_24_ = 1.26, *P* = 0.3; Figure 4C) or water stress (*F*_1_,_24_ = 1.65, *P* = 0.21). The proportions of essential amino acids were not affected by abiotic stresses (temperature rise: *F*_2_,_24_ = 1.47, *P* = 0.25; water stress: *F*_1_,_24_ = 0.83 *P* = 0.37; Figure 4D). However, the relative concentrations of seven amino acids (asparagine, glycine, serine, alanine, isoleucine, valine, and leucine) decreased with temperature rise while the relative concentrations of three other amino acids (phenylalanine, proline, and hydroxyproline) increased with water stress (Figure 4E and Supplementary Table 2). Consequently, the three most abundant amino acids in pollen for plants grown at 27WS were lysine (18.0%), proline (15.8%), and glycine (13.7%).

## Discussion

The effects of temperature rise and water stress on floral resource quantities have been demonstrated (Scaven and Rafferty, 2013; Descamps et al., 2021b) whereas effects on floral resource composition and nutritional quality for pollinators remain largely unknown. Our results showed that temperature rise and water stress influenced both the quantities and compositions of floral resources in the entomophilous species *B*. *officinalis* even if these changes differed according to the studied parameter. While nectar volume, sugar, and amino acid content of nectar were affected mostly by both stresses, pollen quantity, and polypeptide concentration and amino acid content of pollen were affected almost exclusively by temperature rise. For all our studied floral resource parameters, when both temperature rise and water stress were combined, the effects of stresses were additive.

### Nectar Quantity and Sugar Composition

Temperature rise and water stress decreased the overall quantity of nectar with an 80% reduction in nectar volume. These results were consistent with previous observations that temperature rise and water stress decreased nectar volume per flower in *B*. *officinalis* (6.4 ± 1 μl per flower under 21WW, 0.7 ± 0.6 μl per flower under 27WS, Descamps et al., 2018) and in other entomophilous species (Scaven and Rafferty, 2013; Descamps et al., 2021b). The volume of nectar is an evolutionary trade-off between a high volume that is energetically costly and a low volume that would not be sufficient to attract pollinators and ensure an efficient visitation rate of flowers (Faegri and Pijl, 1979; Vaudo et al., 2015; Parachnowitsch et al., 2019). The reduction in volume that we observed in *B*. *officinalis* due to stress may affect pollinator visitation rate and have repercussion on plant reproduction. Physiologically, temperature rise and water stress reduce the quantity and availability of both water and sugars (Lemoine et al., 2013; Lamaoui et al., 2018). The amount of nectar sugars per flower was 3.5 times lower in plants grown in stressful conditions (27WS, 0.57 ± 0.08 mg) compared to control plants (21WW, 3.57 ± 0.28 mg). Decreased sugar quantity affect negatively flight performance of bees (Hendriksma et al., 2014). We also observed that sugar nectar concentration increased with temperature rise, from on average 50°Brix at 21°C to 60°Brix at 24 and 27°C. This increase might perhaps negatively impact nectar uptake by insects as it was recently shown that the opening of the papillae of bee tongue (*Bombus terrestris*) was maximized up to a sugar concentration in the nectar of 55% concentration (Lechantre et al., 2021). A reduced nectar volume per flower decreases also the amount of water that the insects collect through nectar which could influence their water balance, although bees seem less subject to desiccation than most terrestrial insects (Nicolson, 2009).

Floral resource composition, in addition to quantity, determines flower attractiveness for insect pollinators. In our study, the decrease in total sugar content was accompanied by a change in the composition of sugars in the nectar. We observed that water stress decreased the sucrose to hexoses (fructose and glucose) ratio. Relatively few studies have examined the effects of abiotic stresses on the sugar composition in nectar. Similarly to our results, Rering et al. (2020) observed that water stress decreased the proportion of sucrose compared to hexoses in the nectar of *Fagopyrum esculentum*. However, no change in the nectar sugar composition was detected under water stress in other studies (Villarreal and Freeman, 1990 on *Ipomopsis longiflora* and Clearwater et al., 2018 on *Leptospermum scoparium)*. The change in sucrose/hexoses ratio in response to water stress could be explained by a modification in the invertase activity. This enzyme, which catalyzes the cleavage of the disaccharide sucrose into fructose and glucose, is sensitive to nectar sugar concentration (Nepi et al., 2012). According to Rering et al. (2020), nectar sucrose could also be converted into glucose and fructose due to a greater accumulation of reactive oxygen species (ROS) in the nectar of water-stressed plants.

### Pollen Quantity and Polypeptide Concentration

We did not observe a decrease in pollen weight for *B*. *officinalis* in response to water stress, contrarily with other studies (Waser and Price, 2016; Borghi et al., 2019; Rering et al., 2020). By contrast, we observed a 50% decrease of pollen weight due to temperature rise, between 21 and 27°C. The sensitivity of pollen to temperature is relatively well established in the literature (Hedhly, 2011; Mesihovic et al., 2016). For example, a temperature increase of 8°C (22–30°C) reduced the amount of pollen produced by about 30–50% in *Glycine max* (Koti et al., 2005). According to the optimal foraging theory, reduced floral resource quantity per flower increases the visitation cost for pollinators, since they have to visit a greater number of flowers to obtain the same amount of resources (MacArthur and Pianka, 1966; Latty and Trueblood, 2020). The availability of floral resources is a major limiting factor for bee survival (Vaudo et al., 2015; Carvell et al., 2017; Abrahamczyk et al., 2020). Particularly, during summer periods, a shortage of floral resources is observed which could be a cause of bee species extinction (Requier et al., 2017; Balfour et al., 2018; Timberlake et al., 2019; Langlois et al., 2020; Simanonok et al., 2020). Temperature rise combined with drought can reinforce nutritional resource gaps at the landscape level.

While nectar is the main source of carbohydrates for pollinators, pollen is the main source of proteins and amino acids. We observed that temperature rise increased the concentration of polypeptides in the pollen of *B*. *officinalis*. The observed increase in polypeptide concentration in response to temperature rise supports the hypothesis of van der Kooi et al. (2019) which posits that protein and lipid synthesis are stimulated by a temperature increase. The modification of pollen composition in response to abiotic stresses seems, however, to be species-specific. Indeed, this result contrasts with previous observations on another entomophilous species, *Impatiens glandulifera*, grown under the same controlled conditions, where we observed a decrease in pollen polypeptide concentration in response to temperature rise and water stress (Descamps et al., 2021a). To our knowledge, the other study investigating the impact of a temperature increase of 0.5°C compared to ambient temperature under field conditions did not detect any differences on the protein content for *Carduus nutans* (Russo et al., 2020). Although we observed an increase in protein concentration for *B*. *officinalis* pollen under temperature rise (+6°C), this increase was mitigated by a decrease in pollen weight per flower.

### Amino Acid Composition for Both Nectar and Pollen

Both nectar and pollen could be a source of amino acids for pollinators, although the total quantity of amino acids in pollen was more than 1,000 times higher than the quantity of total amino acids in nectar per flower (at 21WW, 0.23 mg in pollen *vs.* 0.19 μg in nectar). The impact of temperature rise and water stress on the amino acid profiles of flower resources differed between nectar and pollen. Total amino acid concentration increased for nectar but not for pollen, while the proportion of essential amino acids decreased in response to temperature rise for nectar but not for pollen. We observed that the total amino acid concentration in the nectar increased while the nectar volume decreased, which is consistent with the observations of Lohaus and Schwerdtfeger (2014) for several species (*Maurandya barclayana*, *Lophospermum erubescens*, and *Brassica napus*). Furthermore, the proportion of essential amino acids decreased in response to temperature rise in nectar but not in pollen.

We observed that the relative proportions of amino acids were modified by temperature rise and water stress for both nectar and pollen in *B*. *officinalis*. Amino acid profiles differ among species, although some phylogenetic trends are detected (Weiner et al., 2010; Ruedenauer et al., 2019b; Jeannerod et al., Submitted). Our results show that intraspecific variations can be observed in response to abiotic stresses. We found that the concentration of proline, in particular, increased with plant stress in both pollen and nectar. Proline is an osmoprotectant that accumulates in plants in response to environmental stresses and acts as a signal to improve stress tolerance (Hayat et al., 2012). Proline is also attractive for insects (Teulier et al., 2016). It contributes to a preferred taste (Alm et al., 1990; Bertazzini et al., 2010). Proline is required for egg-laying by the honey bee queen (Hrassnigg et al., 2003) and it is also used as fuel for the initial phase flight (Carter et al., 2006; Teulier et al., 2016). No other amino acid can be metabolized as rapidly as proline and releases as much ATP without complete metabolism (Carter et al., 2006). The increase in proline in the nectar and pollen of stressed plants could be detected by pollinating insects and, potentially, have positive impacts on them but this needs to be confirmed (Ruedenauer et al., 2019a).

The concentrations of other amino acids were also impacted by plant stress, although the impact of temperature rise was more pronounced than that of water stress. Differences in amino acid composition affect flower visitor behavior, influencing olfactory learning and memory of *Apis mellifera* and bumblebees (Simcock et al., 2014; Ruedenauer et al., 2019a). Recently, it was shown that bumblebee can perceive different amino acid (asparagine, cysteine, glutamic acid, hydroxyproline, lysine, phenylalanine, and serine) by chemotactile information through antennae and even different concentration of a same amino acid (Ruedenauer et al., 2019a). Although the nutrient requirements of honeybees and bumblebees have been partially investigated (Brodschneider and Crailsheim, 2010; Moerman et al., 2017), little is known about the nutrient requirements of wild bees (Woodard and Jha, 2017; Nikkeshi et al., 2021). For pollen, a decrease in the concentration of isoleucine and leucine, two essential amino acids, was observed at elevated temperatures. Bees prefer pollen with high concentration of essential amino acids, particularly isoleucine, leucine, and valine (Cook et al., 2003). We also observed an increase in the phenylalanine proportion (+10% in nectar, +150% in pollen) in response to water stress. Phenylalanine is induced in metabolic pathways of response to water stress (Wan et al., 2021). It was shown that this amino acid was predominant in the nectars of 73 Mediterranean plants (Petanidou et al., 2006), contrary to another study on 30 British species, where this amino acid was present in much lower quantities (Gardener and Gillman, 2001). The predominance of phenylalanine-rich nectar is explained by Petanidou et al. (2006) as a consequence of selection by long-tongue bees due to strong phagostimulatory effect of this amino acid (Inouye and Waller, 1984; Hendriksma et al., 2014). According to our observations, this presence of phenylalanine could also be a consequence of water stress, that plants may experience under a Mediterranean climate. Not all amino acids have the same importance for insects, and there may be species-specific differences in preference. However, there is a lack of information about the specific contribution of each amino acid to insect nutritional requirements.

Our results showed that temperature rise and water stress affect both the quantity and composition of floral resources in a bee-pollinated species. We observed a decrease in the quantity of nectar and pollen and a change in their composition. If the mutualistic relationship is compromised by changes in floral resources, this can affect both partners, plants, and pollinators. For pollinators, the reduction of resources at the flower level may require greater foraging efforts by pollinators for their food supply. Moreover, a modification of the floral resource composition may affect the nutrition and foraging behavior of pollinators. For plants, changes in pollinator foraging behavior can limit the seed set and decrease reproduction. However, both effects of floral resources modifications on pollinator nutrition and behavior and on plant reproduction need to be further investigated.

## Data Availability Statement

The raw data supporting the conclusions of this article will be made available by the authors, without undue reservation.

## Author Contributions

A-LJ initiated the study and obtained funding. CD performed the experiments, analyzed the results, and wrote the first version of the manuscript. All authors discussed the results and contributed to wrote the article and approved the submitted version.

## Conflict of Interest

The authors declare that the research was conducted in the absence of any commercial or financial relationships that could be construed as a potential conflict of interest.

## Publisher’s Note

All claims expressed in this article are solely those of the authors and do not necessarily represent those of their affiliated organizations, or those of the publisher, the editors and the reviewers. Any product that may be evaluated in this article, or claim that may be made by its manufacturer, is not guaranteed or endorsed by the publisher.
