# Supplementary material for: Climate Change–Induced Stress Reduce Quantity and Alter Composition of Nectar and Pollen From a Bee-Pollinated Species (Borago officinalis, Boraginaceae)
Source: Front Plant Sci. 2021 Oct 11;12:755843. doi: 10.3389/fpls.2021.755843 (PMC8542702; doi:10.3389/fpls.2021.755843)
Supplement: Supplementary file 1 [file Data_Sheet_1.docx]

Supplementary Material

**Table S1** : Effects of temperature rise and water stress on nectar amino acid concentrations (ng/mg nectar) of *Borago officinalis* 3 weeks after stress induction.

| Treatment^5^ | 21WW | 21WS | 24WW | 24WS | 27WW | 27WS | Temp^6^ | Water | Temp : Water |
| --- | --- | --- | --- | --- | --- | --- | --- | --- | --- |
| Asparagine^1^ | 1.40 ± 0.37 | 5.15± 2.18 | 3.90 ± 1.55 | 5.92 ± 2.14 | 3.45 ± 1.35 | 5.45 ± 1.45 | F_2,12_=0.54; *P*=0.6 | F_1,12_=3.83; *P*=0.07 | F_2,12_=0.19; *P*=0.82 |
| Glutamine^2^ | 2.08 ± 0.39 | 8.13 ± 2.15 | 6.37 ± 3.52 | 8.90 ± 3.06 | 5.70 ± 2.81 | 9.33 ± 2.38 | F_2,12_=0.57; *P*=0.58 | F_1,12_=3.46; *P*=0.09 | F_2,12_=0.23; *P*=0.8 |
| Cysteine | < LOD | < LOQ | < LOQ | < LOQ | < LOQ | < LOQ | / | / | / |
| Serine | 1.73 ± 0.1 | 5.13 ± 0.91 | 8.93 ± 4.25 | 7.73 ± 1 | 8.98 ± 3.91 | 11.02 ± 1.05 | F_2,12_=3.45; *P*=0.07 | F_1,12_=0.44; *P*=0.52 | F_2,12_=0.42; *P*=0.67 |
| Histidine^3^ | < LOQ | 3.3 ± 0.63 | 4.48 ± 2.04 | 3.95 ± 1.57 | 4.17 ± 0.89 | 7.35 ± 2.6 | F_2,12_=2.34; *P*=0.14 | F_1,12_=1.14; *P*=0.31 | F_1,12_=1.02; *P*=0.39 |
| Glycine | 0.93 ± 0.25 | 1.58 ± 0.39 | 5.6 ± 3.95 | 3.25 ± 1.28 | 8.33 ± 5.82 | 2.67 ± 0.75 | F_2,12_=0.92; *P*=0.43 | F_1,12_=0.86; *P*=0.37 | F_2,12_=0.47; *P*=0.63 |
| Threonine^3^ | < LOQ | 2.01 ± 0.18 | 5.7 ± 2.86 | 3.82 ± 0.68 | 2.6 ± 0.88 | 4.1 ± 0.31 | F_2,12_=3.18; *P*=0.08 | F_1,12_=0.01; *P*=0.91 | F_1,12_=1.03; *P*=0.39 |
| Arginine^3^ | < LOD | 1.48 ± 0.4 | 5.2 ± 1.93 | 3.42 ± 1.18 | 2.8 ± 0.59 | 4.07 ± 1.41 | F_2,12_=8.76; ***P*=0.005** | F_1,12_=0.02; *P*=0.88 | F_1,12_=2.12; *P*=0.16 |
| Methionine^3,4^ | NF | NF | NF | NF | NF | NF | / | / | / |
| Alanine | 1.02 ± 0.11 | 3.07 ± 0.71 | 4.88 ± 1.59 | 3.47 ± 0.43 | 3.53 ± 0.99 | 5.60 ± 0.66 | F_2,12_=4.18; ***P*=0.04** | F_1,12_=1.37; *P*=0.26 | F_2,12_=2.28; *P*=0.15 |
| Tyrosine | < LOD | < LOD | < LOQ | < LOQ | < LOQ | < LOQ | / | / | / |
| Valine^3^ | < LOQ | 1.5 ± 0.39 | 3.58 ± 1.67 | 4.78 ± 1.9 | 3.48 ± 1.13 | 4.22 ± 1.34 | F_2,12_=6.58; ***P*=0.01** | F_1,12_=0.06; *P*=0.94 | F_1,12_=0.06; *P*=0.94 |
| Phenylalanine^3^ | 1.52 ± 0.26 | 3.18 ± 0.37 | 2.33 ± 0.29 | 3.42 ± 0.51 | 2.37 ± 0.32 | 3.68 ± 0.28 | F_2,12_=2.27; *P*=0.15 | F_1,12_=24.87; ***P<*0.001** | F_1,12_=0.39; *P*=0.69 |
| Isoleucine^3^ | < LOD | < LOQ | 1.55 ± 0.3 | < LOQ | < LOQ | 1.83 ± 0.15 | F_2,12_=3.86; *P*=0.05 | F_1,12_=4.26; *P*=0.06 | F_2,12_=0.01; *P*=0.99 |
| Leucine^3^ | < LOD | < LOD | < LOQ | < LOQ | < LOQ | < LOQ | F_2,12_=3.19; *P*=0.07 | F_1,12_=1.37; *P*=0.26 | F_2,12_=0.13; *P*=0.88 |
| Lysine^3^ | NF | NF | NF | NF | NF | NF | / | / | / |
| Proline | 5.35 ± 1.36 | 25.63 ± 6.02 | 32 ± 5.50 | 48.38 ± 3.80 | 31.37 ± 5.54 | 43.45 ± 5.84 | F_2,12_=23.09; ***P*<0.001** | F_1,12_=24.98; ***P*<0.001** | F_2,12_=0.53; *P*=0.60 |
| Hydroxyproline | NF | NF | NF | NF | NF | NF | / | / | / |
| Total quantity | 23.48 ± 2.5 | 66.03 ± 8.87 | 90.37 ± 17.73 | 103.75 ± 7.37 | 83.22 ± 10.25 | 107.45 ± 10.66 | F_2,12_=17.1; ***P*<0.001** | F_1,12_=10.38; ***P=*0.007** | F_2,12_=1.05; *P*=0.38 |
| EAA | 9.33 ± 1.52 | 15.8 ± 1.62 | 26.78 ± 5.32 | 24.2 ± 3.04 | 20.17 ± 3.23 | 28.02 ± 3.75 | F_2,12_=9.82; ***P=*0.003** | F_1,12_=2.24; *P*=0.16 | F_1,12_=1.57; *P*=0.25 |
| nEAA | 14.15 ± 1.71 | 50.23 ± 8.13 | 63.58 ± 12.71 | 79.55 ± 5.33 | 63.05 ± 7.15 | 79.43 ± 7.64 | F_2,12_=20.04; ***P*<0.001** | F_1,12_=15.26; ***P*=0.002** | F_2,12_=1.29; *P*=0.31 |

^1^ Asparagine + aspartic acid ; ^2^ glutamine + glutamic acid ; ^3^ essential amino acids ; ^4^ methionine sulfone ; ^5^N=3 (triplicates), data are means ± SE, 21=21°C, 24=24°C, 27=27°C, WW : well-watered, WS : water-stressed, Temp. : temperature effect ; EAA : essential amino acids (De Groot, 1953) ; nEAA : non essential amino acids ; LOD : limit of detection ; LOQ : limit of quantification ; NF : not found.

**Table S2** : Effects of temperature rise and water stress on pollen amino acid concentrations (mg/g pollen) of *Borago officinalis* 3 weeks after stress induction.

| Treatment^5^ | 21WW | 21WS | 24WW | 24WS | 27WW | 27WS | Temp | Water | Temp : Water |
| --- | --- | --- | --- | --- | --- | --- | --- | --- | --- |
| Asparagine^1^ | 15.42 ± 2.45 | 15.01± 2.33 | 13.49 ± 2.5 | 10.6 ± 1.01 | 7.59 ± 0.77 | 7.92 ± 0.62 | F_2,24_=8.73; ***P*=0.001** | F_1,24_=0.46; *P*=0.51 | F_2,24_=0.46; *P*=0.64 |
| Glutamine^2^ | 24.28 ± 4.15 | 23.45 ± 2.94 | 19.06 ± 1.45 | 16.52 ± 1.85 | 13.78 ± 1.62 | 14.82 ± 1.05 | F_2,24_=8.72; ***P*=0.001** | F_1,24_=0.16; *P*=0.69 | F_2,24_=0.32; *P*=0.73 |
| Cysteine | 2.35 ± 1.19 | 3.65± 2.43 | 1.74 ± 1.09 | 1.41 ± 0.55 | 1.34 ± 0.51 | 1.14 ± 0.55 | F_2,24_=1.19; *P*=0.32 | F_1,24_=0.05; *P*=0.83 | F_2,24_=0.3; *P*=0.75 |
| Serine | 15.2 ± 2.08 | 14.07 ± 1.61 | 12.73 ± 1.45 | 11.84 ± 0.43 | 8.89 ± 0.41 | 10.18 ± 0.91 | F_2,24_=9.45; ***P<*0.001** | F_1,24_=0.1; *P*=0.76 | F_2,24_=0.57; *P*=0.57 |
| Histidine^3^ | 19.32 ± 6.28 | 22.64 ± 9.22 | 13.14 ± 3.71 | 27.66 ± 3.69 | 20.26 ± 1.79 | 21.4 ± 1.98 | F_2,24_=0.01; *P*=0.99 | F_1,24_=2.43; *P*=0.13 | F_2,24_=1.09; *P*=0.35 |
| Glycine | 42.54 ± 12.5 | 37.67 ± 11.58 | 23.94 ± 5.13 | 48.19 ± 3.66 | 36.32 ± 1.31 | 40.61 ± 2.85 | F_2,24_=0.14; *P*=0.87 | F_1,24_=1.54; *P*=0.23 | F_2,24_=1.9; *P*=0.17 |
| Threonine^3^ | 10.38 ± 2.05 | 13.12 ± 3.65 | 8.34± 1.1 | 11.37 ± 2.77 | 10.71 ± 2.23 | 10.68 ± 2.44 | F_2,24_=0.54; *P*=0.59 | F_1,24_=1.7; *P*=0.21 | F_2,24_=0.41; *P*=0.67 |
| Arginine^3^ | 41.29 ± 17.04 | 29.59 ± 6.57 | 16.67 ± 2.99 | 33.84 ± 5.12 | 25.88 ± 3.63 | 31.12 ± 4.66 | F_2,24_=1.25; *P*=0.30 | F_1,24_=0.45; *P*=0.51 | F_2,24_=2.45; *P*=0.11 |
| Methionine^3,4^ | 9.32 ± 0.54 | 15.15 ± 1.85 | 10.38 ± 0.13 | 10.11 ± 0.93 | 9.4 ± 1.31 | NF | F_2,24_=2.47; *P*=0.11 | F_1,24_=1.07; *P*=0.33 | F_2,24_=2.07; *P*=0.15 |
| Alanine | 15.6 ± 3.82 | 14.54 ± 3.05 | 11.83 ± 0.61 | 10.36 ± 1.66 | 9.15 ± 0.83 | 10.38 ± 1.12 | F_2,24_=4.1; ***P*=0.03** | F_1,24_=0.09; *P*=0.76 | F_2,24_=0.27; *P*=0.77 |
| Tyrosine | 3.26 ± 0.4 | 3.07 ± 0.52 | 2.6 ± 0.23 | 2.93 ± 0.5 | 2.77 ± 0.12 | 3.02 ± 0.3 | F_2,24_=0.93; *P*=0.41 | F_1,24_=0.21; *P*=0.65 | F_2,24_=0.42; *P*=0.66 |
| Valine^3^ | 22.03 ± 5.22 | 17.83 ± 5 | 10.18 ± 3 | 9.64 ± 1.38 | 15.68 ± 12.22 | 8.75 ± 0.95 | F_2,24_=2.66; *P*=0.09 | F_1,24_=1.02; *P*=0.32 | F_2,24_=0.24; *P*=0.78 |
| Phenylalanine^3^ | 17.03 ± 5.07 | 18.31 ± 2.74 | 12.52 ± 0.78 | 18.78 ± 2.25 | 16.11 ± 0.85 | 18.99 ± 1.66 | F_2,24_=0.68; *P*=0.52 | F_1,24_=4.65; ***P*=0.04** | F_2,24_=0.85; *P*=0.44 |
| Isoleucine^3^ | 10.14 ± 1.4 | 12.05 ± 1.72 | 8.52 ± 0.73 | 9.05 ± 0.97 | 7.37 ± 0.24 | 7.63 ± 0.87 | F_2,24_=8.32; ***P*=0.002** | F_1,24_=1.45; *P*=0.24 | F_2,24_=0.53; *P*=0.6 |
| Leucine^3^ | 26.82 ± 4.6 | 22.81 ± 2.61 | 16.75 ± 1.15 | 18.63 ± 1.73 | 16.13 ± 0.61 | 18.13 ± 0.87 | F_2,24_=7.74; ***P*=0.003** | F_1,24_=0.01; *P*=0.94 | F_2,24_=1.19; *P*=0.32 |
| Lysine^3^ | 59.82 ± 17.24 | 53.72 ± 13.04 | 41.42 ± 8.91 | 68.81 ± 12.09 | 47.41 ± 6.48 | 54.22 ± 6.32 | F_2,24_=0.2; *P*=0.82 | F_1,24_=1.46; *P*=0.24 | F_2,24_=1.6; *P*=0.22 |
| Proline | 38.63 ± 24.2 | 44.41 ± 27.92 | 22.13 ± 7.12 | 122.77 ± 14.83 | 53.5 ± 4.63 | 53.15 ± 11.24 | F_2,24_=0.6; *P*=0.56 | F_1,24_=4.39; ***P*=0.05** | F_2,24_=4.23; ***P*=0.03** |
| Hydroxyproline | 4.67 ± 2.76 | 7.25 ± 3.49 | 3.25 ± 1.18 | 16.07 ± 2.39 | 8.33 ± 1.11 | 13.14 ± 1.87 | F_2,23_=0.98; *P*=0.39 | F_1,23_=9.53; ***P*=0.005** | F_2,23_=2.55; *P*=0.1 |
| Total | 369.44 ± 76.73 | 351.94 ± 69.86 | 235.99 ± 28.61 | 377.14 ± 53.56 | 279.69 ± 21.86 | 300.19 ± 28.81 | F_2,24_=1.26; *P=*0.3 | F_1,24_=1.65; *P=*0.21 | F_2,24_=1.63; *P*=0.22 |
| EAA | 216.16 ± 40.62 | 199.15 ± 34.89 | 137.91 ± 17.32 | 205.88 ± 18.98 | 168.94 ± 15.23 | 172.32 ± 13.49 | F_2,24_=1.47; *P=*0.25 | F_1,24_=0.83; *P*=0.37 | F_1,24_=1.65; *P*=0.21 |
| nEAA | 153.28 ± 40.16 | 152.8 ± 38.8 | 98.08 ± 12.37 | 171.26 ± 36.03 | 110.75 ± 14.87 | 127. 87± 18.79 | F_2,24_=1.07; *P*=0.36 | F_1,24_=2.74; *P*=0.11 | F_2,24_=1.5; *P*=0.24 |

^1^ Asparagine + aspartic acid ; ^2^ glutamine + glutamic acid ; ^3^ essential amino acids ; ^4^ methionine sulfone ; ^5^N=4 (triplicates), data are means ± SE, 21=21°C, 24=24°C, 27=27°C, WW : well-watered, WS : water-stressed, Temp. : temperature effect ; EAA : essential amino acids (De Groot, 1953) ; nEAA : non essential amino acids ; LOD : limit of detection ; LOQ : limit of quantification ; NF : not found.
